# Supplementary material for: Sulfonylurea Use in Patients with Type 2 Diabetes and COPD: A Nationwide Population-Based Cohort Study
Source: Int J Environ Res Public Health. 2022 Nov 15;19(22):15013. doi: 10.3390/ijerph192215013 (PMC9690079; doi:10.3390/ijerph192215013)
Supplement: Supplementary file 1 [file ijerph-19-15013-s001.zip › ijerph-1983735-supplementary.pdf]

Table S1. Diseases and related ICD-9-CM, ICD-10-CM codes.

Disease	ICD-9-CM codes	ICD-10-CM codes
Type 2 diabetes	250.xx, except 250.1x	E11
Type 1 diabetes	250.1x	E10
Dialysis	V56.0, V56.8, V45.1	Z49.31, Z49.32, Z99.2
Hepatic failure	570, 572.2, 572.4, 572.8	K72.00, K72.01, K72.10, K72.11, K72.90, K76.2, K72.90, K72.91, K76.7, K76.81
Obesity	278.02, 783.1, V85.2, 278.00, 649.1, V77.8, V85.3, 278.01, 649.2, V45.86, V85.4	R63.5, E66.09, E66.1, E66.8, E66.9, Z13.89, E66.01, E66.2
Smoking status	305.1, 649.0, V15.82	F17.200, F17.201, F17.210, F17.220, F17.221, F17.290, F17.291, Z87.891
Alcohol-related disorders	291, 303, 305.0, 571.0-571.3, V11.3, V79.1	F10, K70.40, K70.41, K70.9
Hypertension	401–405, A26	I10, I11, I12, I13, I15, N26
Dyslipidemia	272	E71.30, E71.31, E71.32, E71.39, E75.21, E75.22, E75.23, E75.24, E75.25, E75.29, E75.3, E75.4, E75.5, E75.6, E77, E78.0, E78.1, E78.2, E78.3, E78.4, E78.5, E78.6, E78.70, E78.71, E78.72, E78.79, E78.8, E78.9
Coronary artery disease	410-414	I20, I21, I22, I24, I25.1, I25.2, I25.3, I25.4, I25.5, I25.6, I25.7, I25.81, I25.82, I25.83, I25.84, I25.89, I25.9
Stroke	430-438	G45.0, G45.1, G45.2, G45.3, G45.4, G45.8, G45.9, G46, I60, I61, I62, I63, I65, I66, I67.0, I67.1, I67.2, I67.3, I67.4, I67.5, I67.6, I67.7, I67.8, I67.9, I68, I69
Heart failure	398.91, 402.01, 402.11, 402.91, 404.01, 404.03, 404.11, 404.13, 404.91, 404.93, 428, 429.4	I09.81, I11.0, I13.0, I13.2, I50, I97.0, I97.110, I97.111, I97.120, I97.121, I97.130, I97.131, I97.190, I97.191
Peripheral arterial disease	440.0, 440.20, 440.21, 440.22, 440.23, 440.24, 440.3, 440.4, 443.9, 443.81, 443.89	I70.2, I70.92, I75.0, I73.9
Chronic obstructive pulmonary disease	491, 492, or 496	J41, J42, J44, J43, or J44.9

Chronic kidney disease	250.4x, 403.xx, 404.xx, 585.xx, 586.xx, 581.8x, 791.0x, 593.9x, V42.0x, V45.1x, V56.0x, V56.8x, 39.27, 39.42, 39.43, 39.49, 39.50, 39.53, 39.93, 39.94, or 39.95	E10.2, E10.65, E11.2, E11.65, E13.2, I12, I13, N03, N08, E10.21, E11.21, N05, N06, N07, N14, N15.0, N15.8, N15.9, N16, N17.1, N17.2, N18, N19, Z94.0, Z49.31, Z49.32, Z99.2, Z94.0
Liver cirrhosis	571.5, 571.2, 571.6	K70.2, K70.30, K70.31, K74.0, K74.1, K74.2, K74.60, K74.69, K74.3, K74.4, K74.5
Lung cancer	162.0, 162.2, 162.3, 162.4, 162.5, 162.8, 162.9	C34.00-C34.92
Non-invasive positive pressure ventilation	93.90, 93.91	Z99.81
Invasive mechanical ventilation	96.7	Z99.1
Bacterial pneumonia	480-486	J12-18
Hypoglycemia	251.0x, 251.1x, or 251.2x	E16.0, E16.1, E16.2

Table S2. Hazard ratios (HRs) and 95% confidence intervals (CIs) for death among the sampled patients.

**Variables**	**T2D without sulfonylureas**			**T2D with sulfonylureas**								
	**Event**	**PY**	**IR**	**Event**	**PY**	**IR**	**cHR**	**(95% CI)**	**p-value**	**aHR †**	**(95% CI)**	**p-value**
Sex												
Female	541	17071	31.69	366	20144	18.17	0.58	(0.51, 0.66)	<0.001	0.55	(0.48, 0.63)	<0.001
Male	739	16746	44.13	522	20493	25.47	0.58	(0.52, 0.65)	<0.001	0.51	(0.45, 0.57)	<0.001
Age (years)												
40-49	73	4908	14.87	56	4632	12.09	0.82	(0.58, 1.16)	0.2608	0.6	(0.41, 0.87)	0.0072
50-59	252	12792	19.70	197	15487	12.72	0.66	(0.54, 0.79)	<0.001	0.58	(0.48, 0.7)	<0.001
60-59	394	9955	39.58	264	12873	20.51	0.52	(0.45, 0.61)	<0.001	0.47	(0.4, 0.55)	<0.001
70-80	561	6162	91.04	371	7645	48.53	0.52	(0.45, 0.59)	<0.001	0.51	(0.44, 0.58)	<0.001
Obesity												
No	1270	33085	38.39	883	39745	22.22	0.58	(0.53, 0.63)	<0.001	0.53	(0.48, 0.58)	<0.001
Yes	10	732	13.66	5	892	5.61	0.41	(0.14, 1.19)	0.1004	0.26	(0.02, 3.85)	0.3309
Smoking status												
No	1265	33366	37.91	881	40081	21.98	0.58	(0.53, 0.64)	<0.001	0.53	(0.48, 0.58)	<0.001
Yes	15	450	33.30	7	556	12.60	0.37	(0.15, 0.92)	0.0314	0.11	(0.02, 0.52)	0.0058
CCI												
0	680	26745	25.42	518	30892	16.77	0.67	(0.6, 0.75)	<0.001	0.6	(0.53, 0.67)	<0.001
1	185	3653	50.65	116	4609	25.17	0.49	(0.39, 0.62)	<0.001	0.47	(0.36, 0.6)	<0.001
≥2	415	3418	121.40	254	5136	49.45	0.4	(0.34, 0.47)	<0.001	0.44	(0.37, 0.52)	<0.001
DCSI												
0	347	13891	24.98	297	15570	19.08	0.77	(0.66, 0.9)	<0.001	0.61	(0.52, 0.72)	<0.001
1	207	7499	27.60	135	8550	15.79	0.58	(0.47, 0.72)	<0.001	0.55	(0.44, 0.69)	<0.001
≥2	726	12426	58.42	456	16517	27.61	0.47	(0.42, 0.53)	<0.001	0.47	(0.42, 0.53)	<0.001
OAD numbers												
0-1	875	26205	33.39	738	32074	23.01	0.69	(0.63, 0.76)	<0.001	0.58	(0.53, 0.65)	<0.001
2-3	375	7078	52.98	141	8335	16.92	0.32	(0.27, 0.39)	<0.001	0.35	(0.28, 0.42)	<0.001

>3	30	533	56.25	9	228	39.45	0.73	(0.35, 1.54)	0.4074	0.23	(0.07, 0.81)	0.0222
Insulin												
No	704	22276	31.60	554	25940	21.36	0.68	(0.61, 0.76)	<0.001	0.59	(0.53, 0.66)	<0.001
Yes	576	11541	49.91	334	14697	22.73	0.46	(0.4, 0.52)	<0.001	0.44	(0.39, 0.51)	<0.001

T2D: type 2 diabetes; PY: person-years; IR: incidence rate, per 1,000 person-years; cHR, crude hazard ratio; aHR: adjusted hazard ratio; CCI, Charlson Comorbidity Index; DCSI, Diabetes Complication Severity Index. OAD, oral antidiabetic drug.

aHR [†]: multivariable analysis, including sex, age, comorbidities, medications as shown in Table 1.

Table S3. Hazard ratios (HRs), and 95% confidence intervals (CIs) for cardiovascular events among the sampled patients.

Variables	T2D without sulfonylureas			T2D with sulfonylureas								
	Event	PY	IR	Event	PY	IR	cHR	(95% CI)	p-value	aHR †	(95% CI)	p-value
Sex												
Female	426	15526	27.44	468	18053	25.92	0.94	(0.83, 1.07)	0.3631	0.86	(0.75, 0.98)	0.0224
Male	546	15052	36.27	650	17769	36.58	1	(0.9, 1.12)	0.9479	0.89	(0.79, 1)	0.0497
Age (years)												
40-49	68	4643	14.65	91	4149	21.93	1.5	(1.09, 2.05)	0.0119	1.14	(0.81, 1.61)	0.4444
50-59	213	11976	17.79	312	14087	22.15	1.25	(1.05, 1.49)	0.0122	1.11	(0.93, 1.33)	0.2379
60-59	329	8784	37.45	358	11291	31.71	0.84	(0.72, 0.98)	0.0225	0.78	(0.67, 0.91)	0.0019
70-80	362	5174	69.96	357	6295	56.71	0.8	(0.69, 0.93)	0.0028	0.78	(0.67, 0.91)	0.0015
Obesity												
No	956	29892	31.98	1099	35011	31.39	0.98	(0.9, 1.07)	0.5961	0.88	(0.81, 0.96)	0.0051
Yes	16	686	23.34	19	811	23.43	1.03	(0.53, 2)	0.9335	1.22	(0.55, 2.74)	0.6249
Smoking status												
No	958	30156	31.77	1101	35323	31.17	0.98	(0.9, 1.06)	0.592	0.88	(0.81, 0.96)	0.0045
Yes	14	422	33.18	17	499	34.04	1.03	(0.51, 2.1)	0.9249	0.98	(0.35, 2.79)	0.9758
CCI												
0	560	24666	22.70	697	27812	25.06	1.1	(0.99, 1.23)	0.0825	1	(0.9, 1.13)	0.9337
1	156	3155	49.44	173	3859	44.83	0.89	(0.72, 1.11)	0.3086	0.76	(0.6, 0.95)	0.0161
≥2	256	2756	92.87	248	4150	59.75	0.65	(0.55, 0.78)	<0.001	0.73	(0.61, 0.87)	<0.001
DCSI												
0	237	13127	18.05	300	14337	20.92	1.16	(0.98, 1.38)	0.0857	1.06	(0.89, 1.26)	0.532
1	145	6963	20.83	178	7779	22.88	1.1	(0.88, 1.37)	0.4108	1.03	(0.82, 1.29)	0.794
≥2	590	10488	56.26	640	13705	46.70	0.82	(0.74, 0.92)	<0.001	0.78	(0.7, 0.88)	<0.001
OAD numbers												
0-1	658	23975	27.45	863	28283	30.51	1.11	(1, 1.23)	0.0481	0.96	(0.87, 1.07)	0.4695

2-3	290	6125	47.35	247	7339	33.66	0.7	(0.59, 0.83)	<0.001	0.71	(0.6, 0.85)	<0.001
>3	24	477	50.30	8	201	39.87	0.8	(0.36, 1.78)	0.5802	0.94	(0.32, 2.77)	0.9099
Insulin												
No	557	20360	27.36	672	23127	29.06	1.06	(0.95, 1.19)	0.3105	0.93	(0.83, 1.04)	0.2251
Yes	415	10218	40.62	446	12695	35.13	0.86	(0.75, 0.98)	0.0278	0.82	(0.71, 0.94)	0.0043

T2D: type 2 diabetes; PY: person-years; IR: incidence rate, per 1,000 person-years; cHR, crude hazard ratio; aHR: adjusted hazard ratio; CCI, Charlson Comorbidity Index; DCSI, Diabetes Complication Severity Index. OAD, oral antidiabetic drug.

aHR †: multivariable analysis including sex, age, comorbidities, medications as shown in Table 1.

Table S4. Hazard ratios (HRs), and 95% confidence intervals (CIs) for non-invasive positive pressure ventilation among the sampled patients.

Variables	T2D without sulfonylureas			T2D with sulfonylureas						
	Event	PY	IR	Event	PY	IR	cHR (95% CI)	p-value	aHR † (95% CI)	p-value
Sex										
Female	93	16931	5.49	80	19988	4.00	0.74 (0.55, 1)	0.0528	0.72 (0.53, 0.98)	0.0394
Male	87	16642	5.23	93	20315	4.58	0.87 (0.65, 1.17)	0.3515	0.74 (0.54, 1)	0.049
Age (years)										
40-49	11	4870	2.26	6	4608	1.30	0.58 (0.21, 1.58)	0.2869	0.39 (0.13, 1.2)	0.1022
50-59	27	12761	2.12	38	15415	2.47	1.21 (0.74, 1.99)	0.4406	1.09 (0.65, 1.82)	0.7512
60-59	57	9869	5.78	61	12741	4.79	0.86 (0.6, 1.24)	0.4238	0.87 (0.6, 1.28)	0.4785
70-79	85	6074	14.00	68	7540	9.02	0.61 (0.44, 0.84)	0.0023	0.58 (0.41, 0.8)	0.001
Obesity										

No	-	-		-	-		0.81 (0.66, 1)	0.0553	0.75 (0.6, 0.93)	0.0079
Yes	-	-		-	-		0.44 (0.04, 4.89)	0.5054	- -	-
Smoking status										
No	-	-		-	-		0.82 (0.66, 1.01)	0.0625	0.75 (0.61, 0.94)	0.0102
Yes	-	-		-	-		0.41 (0.08, 2.26)	0.3071	- -	-
CCI										
0	102	26585	3.84	101	30706	3.29	0.88 (0.67, 1.16)	0.3677	0.8 (0.61, 1.07)	0.1309
1	27	3631	7.44	28	4546	6.16	0.83 (0.49, 1.41)	0.4928	0.8 (0.45, 1.45)	0.4661
≥2	51	3357	15.19	44	5051	8.71	0.54 (0.36, 0.81)	0.003	0.65 (0.43, 1)	0.0506
DCSI										
0	42	13834	3.04	53	15480	3.42	1.14 (0.76, 1.71)	0.5263	0.99 (0.65, 1.52)	0.9745
1	21	7470	2.81	32	8487	3.77	1.41 (0.81, 2.44)	0.2256	1.2 (0.67, 2.14)	0.5424

≥2	117	12269	9.54	88	16337	5.39	0.56 (0.43, 0.74)	<0.001	0.56 (0.42, 0.75)	<0.001
OAD numbers										
0-1	-	-	4.30	-	-	4.34	1.01 (0.79, 1.3)	0.9204	0.88 (0.68, 1.14)	0.3441
2-3	-	-	9.00	-	-	4.12	0.48 (0.31, 0.73)	<0.001	0.47 (0.31, 0.73)	<0.001
>3	-	-	9.43	-	-	4.38	0.48 (0.06, 4.12)	0.5023	- -	-
Insulin										
No	101	22115	4.57	101	25772	3.92	0.88 (0.67, 1.16)	0.3714	0.77 (0.58, 1.03)	0.0744
Yes	79	11458	6.89	72	14531	4.95	0.7 (0.51, 0.97)	0.0317	0.72 (0.52, 1)	0.053

T2D: type 2 diabetes; PY: person-years; IR: incidence rate, per 1,000 person-years; cHR, crude hazard ratio; aHR: adjusted hazard ratio; CCI, Charlson Comorbidity Index; DCSI, Diabetes Complication Severity Index. OAD, oral antidiabetic drug.

aHR †: multivariable analysis including sex, age, comorbidities, medications as shown in Table 1.

Table S5. Hazard ratios (HRs), and 95% confidence intervals (CIs) for invasive mechanical ventilation among the sampled patients.

Variables	T2D without sulfonylureas			T2D with sulfonylureas						
	Event	PY	IR	Event	PY	IR	cHR (95% CI)	p-value	aHR † (95% CI)	p-value
Sex										
Female	213	16835	12.65	151	19903	7.59	0.6 (0.49, 0.74)	<0.001	0.57 (0.46, 0.71)	<0.001
Male	292	16478	17.72	238	20124	11.83	0.66 (0.56, 0.79)	<0.001	0.58 (0.49, 0.7)	<0.001
Age (years)										
40-49	32	4883	6.55	40	4564	8.76	1.35 (0.85, 2.15)	0.204	1.04 (0.63, 1.73)	0.8714
50-59	100	12651	7.90	97	15288	6.34	0.81 (0.62, 1.08)	0.1497	0.7 (0.52, 0.93)	0.0151
60-59	175	9775	17.90	111	12679	8.75	0.49 (0.38, 0.62)	<0.001	0.44 (0.34, 0.57	<0.001
70-80	198	6004	32.98	141	7496	18.81	0.54 (0.44, 0.68)	<0.001	0.54 (0.43, 0.67)	<0.001
Obesity										

No	502	32590	15.40	386	39136	9.86	0.64 (0.56, 0.73)	<0.001	0.62 (0.54, 0.7)	<0.001
Yes	3	722	4.15	3	891	3.37	0.83 (0.17, 4.13)	0.8205	0.44 (0.03, 6.04)	0.5356
Smoking status										
No	496	32875	15.09	383	39491	9.70	0.64 (0.56, 0.73)	<0.001	0.58 (0.5, 0.66)	<0.001
Yes	9	438	20.55	6	536	11.20	0.54 (0.19, 1.52)	0.243	0.11 (0.01, 0.83)	0.0325
CCI										
0	297	26378	11.26	224	30544	7.33	0.65 (0.55, 0.77)	<0.001	0.57 (0.47, 0.68)	<0.001
1	69	3609	19.12	60	4502	13.33	0.71 (0.5, 1)	0.049	0.71 (0.49, 1.03)	0.075
≥2	139	3325	41.80	105	4981	21.08	0.49 (0.38, 0.63)	<0.001	0.52 (0.4, 0.68)	<0.001
DCSI										
0	131	13763	9.52	128	15353	8.34	0.88 (0.69, 1.12)	0.2944	0.75 (0.58, 0.97)	0.0284
1	83	7432	11.17	63	8470	7.44	0.68 (0.49, 0.94)	0.0207	0.61 (0.43, 0.87)	0.0054

≥2	291	12118	24.01	198	16204	12.22	0.5 (0.42, 0.6)	<0.001	0.49 (0.4, 0.59)	<0.001
OAD numbers										
0-1	355	25824	13.75	313	31572	9.91	0.72 (0.62, 0.84)	<0.001	0.6 (0.51, 0.7)	<0.001
2-3	143	6957	20.56	73	8233	8.87	0.44 (0.33, 0.58)	<0.001	0.45 (0.33, 0.6)	<0.001
>3	7	532	13.15	3	223	13.45	1.07 (0.28, 4.17)	0.918	3.4 (0, 184559.31)	0.8257
Insulin										
No	294	21960	13.39	236	25564	9.23	0.69 (0.58, 0.82)	<0.001	0.6 (0.5, 0.72)	<0.001
Yes	211	11353	18.59	153	14463	10.58	0.56 (0.46, 0.69)	<0.001	0.53 (0.42, 0.65)	<0.001

T2D: type 2 diabetes; PY: person-years; IR: incidence rate, per 1,000 person-years; cHR, crude hazard ratio; aHR: adjusted hazard ratio; CCI, Charlson Comorbidity Index; DCSI, Diabetes Complication Severity Index. OAD, oral antidiabetic drug.

aHR †: multivariable analysis including sex, age, comorbidities, medications as shown in Table 1.

Table S6. Hazard ratios (HRs), and 95% confidence intervals (CIs) for bacterial pneumonia among the sampled patients.

Variables	T2D without sulfonylureas			T2D with sulfonylureas						
	Event	PY	IR	Event	PY	IR	cHR (95% CI)	p-value	aHR † (95% CI)	p-value
Sex										
Female	324	16076	20.15	344	18972	18.13	0.9 (0.78, 1.05)	0.1948	0.84 (0.72, 0.98)	0.0262
Male	410	15651	26.20	404	19280	20.95	0.8 (0.69, 0.91)	0.0012	0.73 (0.63, 0.84)	<0.001
Age (years)										
40-49	45	4742	9.49	52	4424	11.75	1.23 (0.83, 1.84)	0.3034	1 (0.64, 1.54)	0.9855
50-59	173	12162	14.23	188	14847	12.66	0.9 (0.73, 1.11)	0.32	0.83 (0.67, 1.03)	0.0887
60-59	236	9248	25.52	232	12097	19.18	0.76 (0.63, 0.91)	0.0024	0.73 (0.61, 0.88)	<0.001
70-80	280	5575	50.23	276	6884	40.09	0.77 (0.65, 0.91)	0.0019	0.72 (0.61, 0.86)	<0.001
Obesity										

No	721	31027	23.24	742	37398	19.84	0.86 (0.77, 0.95)	0.0028	0.79 (0.71, 0.88)	<0.001
Yes	13	700	18.58	6	854	7.03	0.36 (0.14, 0.95)	0.0388	0.29 (0.08, 1.01)	0.0519
Smoking status										
No	727	31280	23.24	736	37738	19.50	0.84 (0.76, 0.93)	<0.001	0.78 (0.7, 0.87)	<0.001
Yes	7	447	15.66	12	514	23.35	1.46 (0.57, 3.71)	0.4258	2.59 (0.49, 13.83)	0.265
CCI										
0	488	25182	19.38	482	29310	16.44	0.85 (0.75, 0.97)	0.0148	0.77 (0.67, 0.87)	<0.001
1	98	3421	28.65	92	4275	21.52	0.74 (0.56, 0.98)	0.0368	0.76 (0.56, 1.03)	0.0726
≥2	148	3125	47.37	174	4667	37.28	0.77 (0.61, 0.95)	0.0177	0.86 (0.68, 1.08)	0.2025
DCSI										
0	211	13289	15.88	234	14890	15.71	1 (0.83, 1.2)	0.982	0.85 (0.7, 1.03)	0.0921
1	142	6986	20.33	137	8112	16.89	0.84 (0.66, 1.06)	0.1429	0.76 (0.6, 0.97)	0.0279

≥2	381	11452	33.27	377	15250	24.72	0.74 (0.64, 0.85)	<0.001	0.74 (0.64, 0.86)	<0.001
OAD numbers										
0-1	546	24598	22.20	582	30221	19.26	0.87 (0.77, 0.97)	0.015	0.76 (0.67, 0.86)	<0.001
2-3	171	6637	25.77	163	7807	20.88	0.82 (0.66, 1.02)	0.0803	0.88 (0.71, 1.11)	0.2809
>3	17	493	34.51	3	224	13.39	0.39 (0.11, 1.33)	0.1313	0.05 (0, 0.61)	0.0199
Insulin										
No	463	20830	22.23	434	24618	17.63	0.8 (0.7, 0.91)	<0.001	0.71 (0.62, 0.81)	<0.001
Yes	271	10897	24.87	314	13634	23.03	0.92 (0.78, 1.08)	0.3091	0.91 (0.77, 1.08)	0.2784

T2D: type 2 diabetes; PY: person-years; IR: incidence rate, per 1,000 person-years; cHR, crude hazard ratio; aHR: adjusted hazard ratio; CCI, Charlson Comorbidity Index; DCSI, Diabetes Complication Severity Index. OAD, oral antidiabetic drug.

aHR †: multivariable analysis including sex, age, comorbidities, medications as shown in Table 1.
